# Genetic analysis of *LRRK2* variants in Han Chinese patients with Parkinson’s disease

**DOI:** 10.1371/journal.pone.0340448

**Published:** 2026-01-08

**Authors:** Xinyue Deng, Xue Yan, Zhi Song, Wen Zheng, Hongbo Xu, Yan Yang, Jiangang Wang, Lamei Yuan, Hao Deng

**Affiliations:** 1 Health Management Center, the Third Xiangya Hospital, Central South University, Changsha, China; 2 Center for Experimental Medicine, the Third Xiangya Hospital, Central South University, Changsha, China; 3 Disease Genome Research Center, Central South University, Changsha, China; 4 Xiangya School of Medicine, Central South University, Changsha, China; 5 Department of Neurology, the Third Xiangya Hospital, Central South University, Changsha, China; 6 Research Center of Medical Experimental Technology, the Third Xiangya Hospital, Central South University, Changsha, China; Institute for Clinical Epidemiology and Applied Biometry, GERMANY

## Abstract

Parkinson’s disease (PD) is the second most common neurodegenerative disorder, with variants in the leucine rich repeat kinase 2 gene (*LRRK2*) being frequent genetic causes of inherited PD. This study aimed to screen for *LRRK2* variants and assess their effects on PD susceptibility in the Han Chinese population. *LRRK2* variants were identified through whole exome sequencing and confirmed by Sanger sequencing in 468 unrelated PD patients and 566 controls. The identified variants were analyzed via bioinformatics predictions and statistical analyses, by interpreting existing literature and database evidence. Twelve variants were detected, including p.A419V, p.P755L, p.I786F, p.C925Y, p.M968K, p.R1067Q, p.R1320S, p.I1339M, p.P1446L, p.D1756Y, p.H2206Y, and p.G2385R. Among participants, 14.74% of PD cases and 7.24% of controls carried at least one *LRRK2* variant (odds ratio [OR]: 2.2144, 95% confidence interval [CI]: 1.4728–3.3294, *P* = 0.0001). The residues p.P755, p.R1067, p.R1320, p.P1446, and p.D1756 are comparatively conserved. The variants p.R1067Q and p.D1756Y, absent in controls, were predicted to be damaging. Compared to controls, PD patients had higher frequencies of p.A419V (OR: 4.2820, 95% CI: 1.4047–13.0530, *P* = 0.0054, corrected *P* = 0.0324) and p.G2385R (OR: 2.1149, 95% CI: 1.2682–3.5268, *P* = 0.0034, corrected *P* = 0.0324). These findings suggest that the *LRRK2* variants, p.R1067Q and p.D1756Y, may act as likely pathogenic variants in PD, while p.A419V and p.G2385R might be risk factors for increased PD susceptibility in the Han Chinese population.

## Introduction

Parkinson’s disease (PD) is the second most common progressive neurodegenerative disorder, with an estimated global prevalence ranging from 1.52‰ in 2021 to 2.67‰ by 2050, primarily due to significant changes in age structure, industrialization, urbanization, and socioeconomic development [1,2]. Clinically, PD is typically manifested as a movement disorder with cardinal motor symptoms, including bradykinesia, rest tremor, rigidity, and postural instability [2,3]. Additionally, other motor symptoms and various non-motor features, such as sensory dysfunction, sleep disturbances, cognitive decline, psychiatric abnormalities, and autonomic dysfunction, are common and also affect patients’ quality of life [4,5]. The neuropathological hallmarks of PD are selective dopaminergic neuronal loss in the substantia nigra pars compacta of the ventral midbrain and the aggregation of the synaptic vesicle-associated protein alpha-synuclein in Lewy bodies [4,6,7]. Current therapeutic strategies are centered on dopamine replacement therapy, along with other pharmacological and non-pharmacological interventions, and emerging personalized approaches, which hold great potential to more effectively alleviate symptoms and better individualize therapy [5,8]. Besides advanced age as a contributing factor, genetic factors, environmental influences, and their interactions indisputably play important roles in the susceptibility, development, and progression of PD [8].

Variants in the leucine rich repeat kinase 2 gene (*LRRK2*) have been clearly identified as the most frequent genetic cause of inherited PD [9]. The prevalence of *LRRK2* variants in PD seemingly presents marked heterogeneity across populations with different regional and ethnic backgrounds [10]. The vast majority of genetic studies concerning PD have been rooted in populations of European descent, leading to the possibility that many risk variants, particularly those specific to underrepresented populations, are likely underestimated or remain uncovered [11]. This case-control study aimed to screen *LRRK2* variants and evaluate their risk effects on the susceptibility to PD in Han Chinese. This study replicated and compiled previous genetic discoveries and may provide new insights into the potential differences in *LRRK2* variants’ contributions to PD susceptibility among diverse populations.

## Materials and methods

### Study subjects

The cohort consisted of 468 unrelated PD patients [mean age at sampling: 58.37 ± 10.96 years, mean onset age: 54.92 ± 11.15 years, 242 males (51.71%) and 226 females (48.29%)] and 566 age- and sex-matched controls [mean age at sampling: 60.85 ± 8.80 years, 300 males (53.00%) and 266 females (47.00%)]. All subjects were of Han Chinese origin and recruited at the Third Xiangya Hospital, Central South University, China, from 26/12/2018–12/05/2023, without overlap with the previously reported cohort by Zhao et al [12]. All enrolled individuals provided written informed consent prior to participation. Among them, 400 PD patients were analyzed in a previous study showing no variants in the synuclein alpha gene (*SNCA*) [13]. PD patients were diagnosed by two experienced neurologists according to the diagnostic criteria recommended by the International Parkinson and Movement Disorder Society [3]. Our study was conducted from December 2018 to May 2023, in accordance with the Helsinki Declaration and approved by the Institutional Review Board of the Third Xiangya Hospital, Central South University.

### Variant analysis

Genomic DNA samples from all participants were isolated from peripheral blood leukocytes using the established standard phenol-chloroform extraction procedure. Whole exome sequencing (WES) was performed on 468 PD patients and 566 controls by BGI-Shenzhen, China, as previously mentioned [14]. Exome capture and enrichment utilized the Agilent SureSelect Human All Exon V6, and high-throughput sequencing was conducted on the BGISEQ-500 platform. Following data filtering, the clean data were aligned to the human reference genome (GRCh37/hg19). The mean sequencing coverage achieved a depth of at least 100 × , with more than 99% of targeted bases covered and over 90% covered at a depth of ≥20 × . Variant calling was performed to identify single nucleotide polymorphisms and insertions-deletions, followed by annotation. Additionally, copy number variants (CNVs) were analyzed using the CNVnator (version 0.3.2) read-depth algorithm in 100 PD cases [13]. The analysis focused on CNVs in established PD genes such as *LRRK2*, *SNCA*, the Parkinsonism associated deglycase gene (*PARK7*), the PTEN induced kinase 1 gene (*PINK1*), and the parkin RBR E3 ubiquitin protein ligase gene (*PRKN*). The identified *LRRK2* variants were confirmed by Sanger sequencing using paired primers designed by Primer3 (https://primer3.ut.ee/, S1 Table). NCBI Basic Local Alignment Search Tool (BLAST, https://blast.ncbi.nlm.nih.gov/Blast.cgi) was used to evaluate the evolutionary conservation of altered amino acids across ten species (zebrafish, tropical clawed frog, chicken, Norway rat, house mouse, cattle, dog, Rhesus monkey, chimpanzee, and human). According to the minor allele frequencies (MAFs) from East Asian cohorts in the Genome Aggregation Database (gnomAD, v2.1.1), the Exome Aggregation Consortium (ExAC, v1.0), and the 1000 Genomes Project (1kGP, Phase 3), the detected variants were classified into three categories: rare (MAFs < 0.001), low frequency (0.001 ≤ MAFs ≤ 0.01), and common (MAFs > 0.01). The pathogenicity of each identified *LRRK2* variant was predicted using several bioinformatics prediction tools. Variants were considered “damaging” if supported by at least three of five tools, including Protein Variation Effect Analyzer (predicted as “deleterious”), Polymorphism Phenotyping v2 (predicted as “probably damaging” or “possibly damaging”), and Sorting Intolerant from Tolerant (predicted as “damaging”), Combined Annotation Dependent Depletion (v1.7, phred score > 15), and Rare Exome Variant Ensemble Learner (score > 0.5) [15–19]. Variants were clinically annotated with ClinVar (https://www.ncbi.nlm.nih.gov/clinvar/) and the Human Gene Mutation Database (HGMD, https://www.hgmd.cf.ac.uk/ac/index.php). They were further searched in PubMed (https://pubmed.ncbi.nlm.nih.gov/) to determine whether they were previously reported in pedigrees or sporadic cases, as well as in the Movement Disorder Society Genetic mutation database (MDSGene, https://www.mdsgene.org/) [20,21].

### Statistical analysis

The identified *LRRK2* variants were statistically analyzed using SPSS software (version 26, SPSS Inc., Chicago, IL, USA). Pearson’s chi-squared test or Fisher’s exact test was applied to compare the distributions of genotypic and allelic frequencies between the case and control groups. The Hardy-Weinberg equilibrium was tested. The false discovery rate (FDR) correction was used for multiple comparisons using Benjamini-Hochberg method and the corrected *P* value < 0.05 was considered statistically significant. Post-hoc power calculations were performed using the Power and Sample Size Calculations (version 3.1.6) [22]. The statistical analysis results, combined with evolutionary conservation, MAFs, predicted pathogenicity, and curated evidence from MDSGene, were used to assess the potential pathogenicity of the detected variants.

## Results

**Fig 1:**
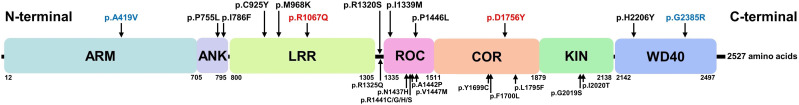
The schematic diagram of the LRRK2 protein with different functional domains [9]. Reported definitive disease-associated variants are listed below the diagram. The variants detected in this study are listed above the diagram, of which the considered likely pathogenic variants and risk variants are highlighted in red and blue, respectively. ANK, ankyrin; ARM, armadillo; COR, C-terminal of ROC; KIN, kinase; LRR, leucine-rich repeat; LRRK2, leucine rich repeat kinase 2; ROC, ras-of-complex.

The WES data revealed twelve *LRRK2* missense variants (NM_198578.4, NP_940980.4, Fig 1), including c.1256C > T (p.A419V, rs34594498), c.2264C > T (p.P755L, rs34410987), c.2356A > T (p.I786F, rs544929315), c.2774G > A (p.C925Y, rs201810995), c.2903T > A (p.M968K, rs750535941), c.3200G > A (p.R1067Q, rs111341148), c.3960G > T (p.R1320S, rs77018758), c.4017T > G (p.I1339M, rs773070538), c.4337C > T (p.P1446L, rs74681492), c.5266G > T (p.D1756Y, rs538052823), c.6616C > T (p.H2206Y, rs770591392), and c.7153G > A (p.G2385R, rs34778348). Two variants, p.M968K and p.H2206Y, have not been previously reported in the literature. No explanatory pathogenic CNVs were detected in 100 PD cases. All identified *LRRK2* missense variants were successfully confirmed by Sanger sequencing (S1 Fig).

**Fig 2:**
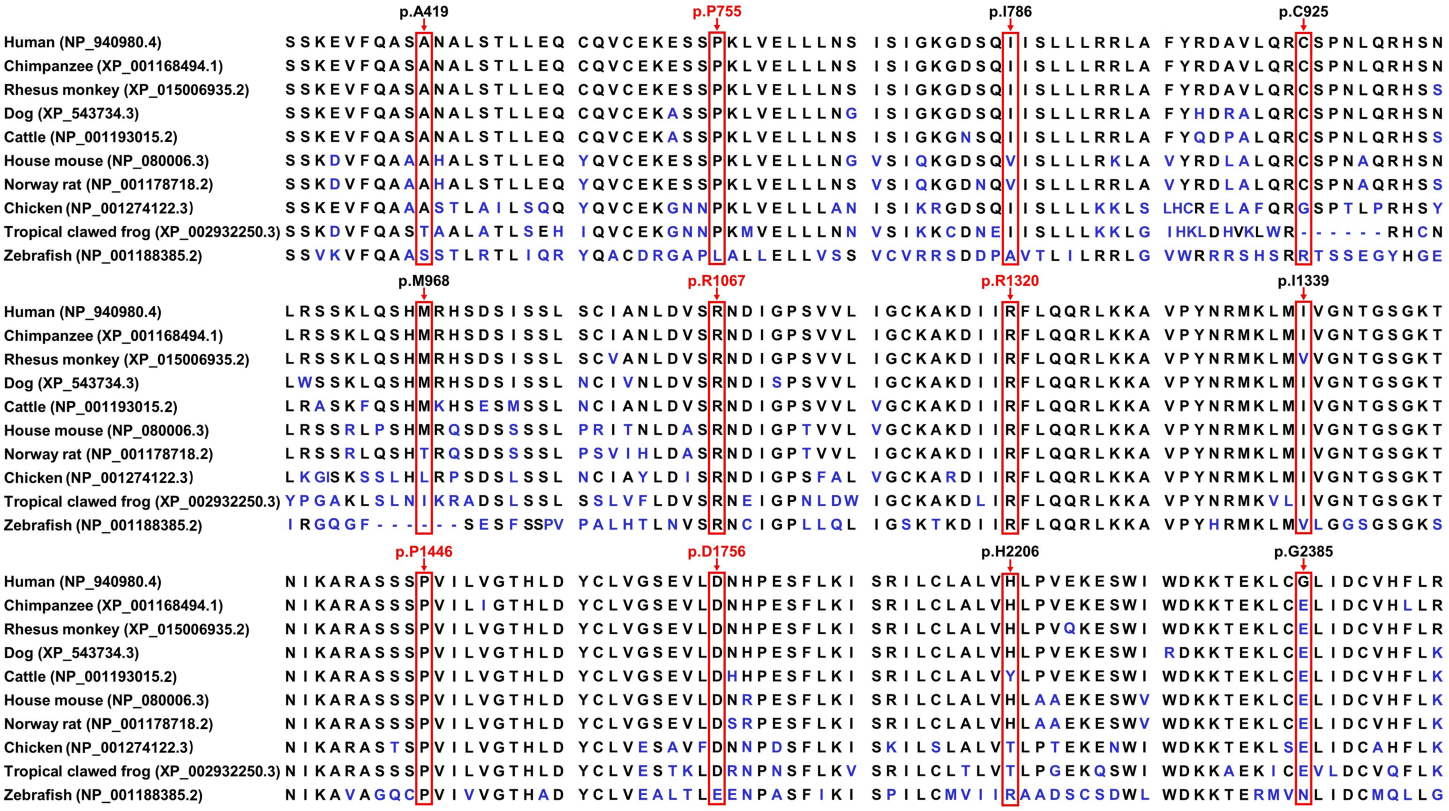
Conservation analyses of partial LRRK2 protein regions. The arrows indicate the amino acid residues at the mutated sites and those conserved in at least nine of ten species are highlighted in red. LRRK2, leucine rich repeat kinase 2.

The residues p.P755, p.R1067, p.R1320, p.P1446, and p.D1756 are conserved in at least nine of the ten species, while p.G2385 is not conserved among humans and other species, in which glutamic acid is common in most species (Fig 2). The variants p.I786F, p.M968K, p.R1067Q, p.I1339M, p.D1756Y, and p.H2206Y with MAFs < 0.001 or absent in East Asian cohorts of gnomAD, ExAC, and 1kGP were considered as rare variants. The variants p.A419V, p.P755L, p.C925Y, p.R1320S, and p.P1446L were classified as low-frequency variants (0.001 ≤ MAFs ≤ 0.01), while p.G2385R was classified as a common variant (MAFs > 0.01). The variants p.A419V, p.I786F, p.R1067Q, p.I1339M, p.P1446L, and p.D1756Y were predicted to be damaging based on combined in silico predictions. The variants p.M968K and p.H2206Y were absent from both ClinVar and HGMD. The MAFs and potential pathogenicity of the detected *LRRK2* missense variants are listed in Table 1.

**Table 1:** Minor allele frequencies and potential pathogenicity of twelve detected *LRRK2* missense variants.

Variants	dbSNP	Domain	MAFs						Bioinformatics predictions^b^					Pathogenicity
			gnomAD v2.1.1		ExAC v1.0		1kGP Phase 3		PROVEAN	PolyPhen-2 HumDiv	SIFT	CADD	REVEL	
			Total	EAS	Total	EAS	Total	EAS						
**c.1256C > T (p.A419V)** ^**a**^	rs34594498	ARM	0.000485	0.004813	0.000514	0.005796	0.001398	0.006944	Neutral	**Probably damaging**	**Damaging**	**25.0**	0.175	**Risk**
**c.2264C > T (p.P755L)**	rs34410987	ANK	0.000701	0.009293	0.000693	0.009141	0.001597	0.006944	Neutral	Benign	Tolerated	**22.0**	0.173	Likely benign
**c.2356A > T (p.I786F)**	rs544929315	ANK	0.000053	0.000752	0.000033	0.000462	0.000200	0.000992	**Deleterious**	**Possibly damaging**	**Damaging**	**15.58**	0.095	Uncertain
**c.2774G > A (p.C925Y)**	rs201810995	LRR	0.000082	0.001053	0.000075	0.001047	0.000399	0.001984	Neutral	Benign	Tolerated	11.88	0.039	Likely benign
**c.2903T > A (p.M968K)**	rs750535941	LRR	0.000039	0.000551	0.000041	0.000579	ND	ND	Neutral	Benign	**Damaging**	12.28	0.055	Uncertain
**c.3200G > A (p.R1067Q)** ^**a**^	rs111341148	LRR	0.000032	0.000200	0.000033	0.000231	ND	ND	Neutral	**Probably damaging**	**Damaging**	**28.4**	0.282	**Likely pathogenic**
**c.3960G > T (p.R1320S)**	rs77018758	–	0.000150	0.002011	0.000174	0.002447	0.000998	0.004960	Neutral	Benign	Tolerated	**25.3**	0.319	Likely benign
**c.4017T > G (p.I1339M)**	rs773070538	ROC	0.000012	0.000000	0.000008	0.000000	ND	ND	Neutral	**Probably damaging**	**Damaging**	**21.4**	**0.585**	Uncertain
**c.4337C > T (p.P1446L)**	rs74681492	ROC	0.000184	0.002557	0.000247	0.003352	0.000998	0.004960	**Deleterious**	**Probably damaging**	**Damaging**	**28.3**	**0.832**	Uncertain
**c.5266G > T (p.D1756Y)** ^**a**^	rs538052823	COR	0.000021	0.000301	0.000008	0.000116	0.000200	0.000992	Neutral	**Probably damaging**	**Damaging**	**26.2**	**0.531**	**Likely pathogenic**
**c.6616C > T (p.H2206Y)**	rs770591392	WD40	0.000004	0.000054	ND	ND	ND	ND	Neutral	Benign	Tolerated	12.51	0.085	Uncertain
**c.7153G > A (p.G2385R)** ^**a**^	rs34778348	WD40	0.001680	0.022376	0.001584	0.020654	0.004792	0.023810	Neutral	Benign	Tolerated	**22.2**	0.044	**Risk**

ANK, ankyrin; ARM, armadillo; CADD, Combined Annotation Dependent Depletion; COR, C-terminal of ROC; dbSNP, the Single Nucleotide Polymorphism database; EAS, East Asians; ExAC, the Exome Aggregation Consortium; gnomAD, the Genome Aggregation Database; LRR, leucine-rich repeat; *LRRK2*, the leucine rich repeat kinase 2 gene; MAFs, minor allele frequencies; ND, no data; PolyPhen-2, Polymorphism Phenotyping v2; PROVEAN, Protein Variation Effect Analyzer; REVEL, Rare Exome Variant Ensemble Learner; ROC, ras-of-complex; SIFT, Sorting Intolerant from Tolerant; VUS, variant of uncertain significance; 1kGP, the 1000 Genomes Project.

^a^The variant is categorized as a likely pathogenic or risk variant, where its corresponding pathogenicity interpretations are shown in bold font.

^b^Bold text indicates supporting evidence of variant pathogenicity (predicted deleterious by PROVEAN, PolyPhen-2, and SIFT, CADD phred score > 15, and REVEL score > 0.5).

The Hardy-Weinberg equilibrium test showed no deviation in any of the variants for the controls (*P* > 0.05). Among the total participants, about 14.74% (69/468) of PD cases and 7.24% (41/566) of controls were found to carry at least one of these variants (odds ratio [OR]: 2.2144, 95% confidence interval [CI]: 1.4728–3.3294, *P* = 0.0001). In the case group, one individual carried heterozygous p.A419V and p.G2385R, and another carried heterozygous p.I786F and p.G2385R, simultaneously. All the identified variants were in a heterozygous state, except for p.A419V and p.D1756Y, which were homozygous in a 62-year-old male with an onset age of 58 and a 77-year-old female with an onset age of 77, respectively. In this cohort, the *LRRK2* p.R1067Q variant was identified in an early-onset PD patient with no family history. The male patient exhibited rigidity, rest tremor, and hypertonia at the age 40. He showed bradykinesia, rigidity, tremor, gait difficulty, postural instability, and obvious asymmetry, as well as a clear response to dopamine replacement therapy. Besides, he suffered from non-motor symptoms such as constipation and hyposmia. By reviewing the available data in PubMed and MDSGene, motor characteristics of PD, including bradykinesia (16/17, 94.12%), rigidity (15/16, 93.75%), tremor (13/18, 72.22%), gait difficulty (8/9, 88.89%), and postural instability (7/12, 58.33%), often with obvious asymmetry (8/10, 80%), were frequently observed in 24 p.R1067Q carriers (including 23 previously reported cases and our patient) [23–31]. These patients generally demonstrated a fine response to dopamine, as observed in our case. Non-motor features were various, with constipation being the most frequent symptom.

Compared to controls, PD patients had higher frequencies of two variants, p.A419V (T *vs.* C allele, OR: 4.2820, 95% CI: 1.4047–13.0530, *P* = 0.0054, FDR-corrected *P* = 0.0324) and p.G2385R (A *vs.* G allele, OR: 2.1149, 95% CI: 1.2682–3.5268, *P* = 0.0034, FDR-corrected *P* = 0.0324). The statistical power for the p.A419V and p.G2385R variants in our sample size was estimated to exceed 0.7 at an α level of 0.05 (0.778 and 0.827, respectively), whereas the power for the remaining variants was below 0.5. In contrast, no significant differences were found between the case and control groups regarding p.P755L, p.I786F, p.C925Y, p.M968K, p.R1067Q, p.R1320S, p.I1339M, p.P1446L, p.D1756Y, and p.H2206Y (all *P *> 0.05, Table 2).

**Table 2:** Allelic and genotypic distributions of twelve detected *LRRK2* missense variants in Han Chinese patients with PD and controls.

Variants	Allele	Cases (n = 936)	Controls (n = 1132)	*P* value	OR (95% CI)	Genotype	Cases (n = 468)	Controls (n = 566)	*P* value
**c.1256C > T (p.A419V)** ^**a**^	C	922	1128	**0.0054**	4.2820 (1.4047–13.0530)	CC	455	562	**0.0146**
	T	14	4			CT	12	4	
	–	–	–	–	–	TT	1	0	
**c.2264C > T (p.P755L)**	C	929	1123	0.9030	0.9402 (0.3488–2.5342)	CC	461	557	0.9026
	T	7	9			CT	7	9	
	–	–	–	–	–	TT	0	0	
**c.2356A > T (p.I786F)**	A	935	1132	0.4526	–	AA	467	566	0.4526
	T	1	0			AT	1	0	
	–	–	–	–	–	TT	0	0	
**c.2774G > A (p.C925Y)**	G	935	1131	1.0000	1.2096 (0.0756–19.3651)	GG	467	565	1.0000
	A	1	1			GA	1	1	
	–	–	–	–	–	AA	0	0	
**c.2903T > A (p.M968K)**	T	935	1132	0.4526	–	TT	467	566	0.4526
	A	1	0			TA	1	0	
	–	–	–	–	–	AA	0	0	
**c.3200G > A (p.R1067Q)**	G	935	1132	0.4526	–	GG	467	566	0.4526
	A	1	0			GA	1	0	
	–	–	–	–	–	AA	0	0	
**c.3960G > T (p.R1320S)**	G	934	1130	1.0000	1.2099 (0.1701–8.6053)	GG	466	564	1.0000
	T	2	2			GT	2	2	
	–	–	–	–	–	TT	0	0	
**c.4017T > G (p.I1339M)**	T	935	1132	0.4526	–	TT	467	566	0.4526
	G	1	0			TG	1	0	
	–	–	–	–	–	GG	0	0	
**c.4337C > T (p.P1446L)**	C	935	1131	1.0000	1.2096 (0.0756–19.3651)	CC	467	565	1.0000
	T	1	1			CT	1	1	
	–	–	–	–	–	TT	0	0	
**c.5266G > T (p.D1756Y)**	G	934	1132	0.2047	–	GG	467	566	0.4526
	T	2	0			GT	0	0	
	–	–	–	–	–	TT	1	0	
**c.6616C > T (p.H2206Y)**	C	935	1132	0.4526	–	CC	467	566	0.4526
	T	1	0			CT	1	0	
	–	–	–	–	–	TT	0	0	
**c.7153G > A (p.G2385R)** ^**a**^	G	895	1108	**0.0034**	2.1149 (1.2682–3.5268)	GG	427	542	**0.0029**
	A	41	24			GA	41	24	
	–	–	–	–	–	AA	0	0	

*LRRK2*, the leucine rich repeat kinase 2 gene; OR (95% CI), odds ratio with 95% confidence interval; PD, Parkinson’s disease.

^a^The variant has statistical significance after multiple testing corrections (FDR-corrected *P* < 0.05), where the corresponding nominal *P* value is shown in bold font.

The rare variants p.R1067Q and p.D1756Y, involving highly conserved residues and predicted damaging, were considered as likely pathogenic variants. This interpretation is consistent with the classification of p.R1067Q in MDSGene. The variants p.A419V and p.G2385R with higher frequencies in PD cases than in controls were considered as risk variants. The rare variants p.I786F and p.I1339M, predicted damaging but implicating the not conserved residues, and the low-frequency variant p.P1446L predicted damaging and involving a highly conserved residue, were considered variants of uncertain significance. The rare variants p.M968K and p.H2206Y neither predicted damaging nor implicating conserved residues, were also classified as variants of uncertain significance. The remaining variants, p.P755L, p.C925Y, and p.R1320S, which were neither rare nor predicted damaging, were considered as likely benign variants.

## Discussion

Well-established and robust genetic evidence has identified several unequivocal causative genes for PD, such as *SNCA*, *LRRK2*, *VPS35* (the VPS35 retromer complex component gene), *PRKN*, *PINK1*, and *PARK7* [7]. Since the first identification of the *LRRK2* variant as a cause of PD in 2004, it has been under study for two decades, accounting for at least 5% of familial cases and approximately 1%–2% idiopathic forms of the disease [32,33].

The *LRRK2* gene was originally mapped to chromosome 12p11.2-q13.1 through a genome-wide linkage investigation of a Japanese pedigree with autosomal dominant Parkinsonism in Sagamihara City in 2002 [34]. LRRK2 is ubiquitously expressed in various tissues, including brain regions, lung, kidney, intestine, and immune system (Human Protein Atlas, https://www.proteinatlas.org/) [32,35,36]. The *LRRK2* gene contains 51 exons and encodes a large ~286-kDa protein of 2527 amino acids with multiple domains and functions, which predominantly exists as an active dimer [37,38]. The evolutionarily conserved ras-of-complex (ROC) followed by the C-terminal of ROC (COR) and the abutting tyrosine kinase-like kinase (KIN) constitute a “catalytic core” related to GTPase and kinase activities, respectively [9,39]. The remaining domains, N-terminal armadillo (ARM), ankyrin (ANK), leucine-rich repeat (LRR), and C-terminal WD40, are protein-protein interaction domains [9].

The *LRRK2* gene variants have different frequencies, penetrance, deleteriousness, and effects, ranging from rare variants with high penetrance that dramatically increase PD risk and often cause a monogenic form, to more common variants that slightly enhance risk or exert protective roles [6,40,41]. The availability of next-generation sequencing, such as WES and whole genome sequencing, and advances in downstream LRRK2 biomarkers have been key drivers of *LRRK2*-associated PD research [42,43]. In this study, twelve *LRRK2* missense variants, p.A419V, p.P755L, p.I786F, p.C925Y, p.M968K, p.R1067Q, p.R1320S, p.I1339M, p.P1446L, p.D1756Y, p.H2206Y, and p.G2385R, were identified in 14.74% of PD cases and 7.24% of control subjects. Enrichment analyses, including conservation analyses, MAFs, predictive algorithms, MDSGene criteria, and statistical evaluations, indicated that p.A419V, p.R1067Q, p.D1756Y, and p.G2385R may have potential effects on PD. The rare variants p.R1067Q and p.D1756Y, involving high conserved residues and predicted to be damaging, were considered as likely pathogenic variants. Compared to the controls, PD patients exhibited elevated frequencies of two variants (FDR-corrected *P* < 0.05), p.A419V and p.G2385R, suggesting that these variants may act as risk factors for PD. Variants p.I786F, p.M968K, p.I1339M, p.P1446L, and p.H2206Y were classified as variants of uncertain significance. The variants p.P755L, p.C925Y, and p.R1320S, which were neither rare nor predicted pathogenic, were deemed likely benign. Notably, common missense *LRRK2* variants such as p.N551K (rs7308720) and p.R1628P (rs33949390) were not identified in our cohort, which may result from the limited sample size, age and ethnic stratification, and population-specific genetic constitution.

Two rare variants, p.R1067Q and p.D1756Y, found in two patients but absent in our control group, may be potential disease-associated variants with incomplete penetrance. The p.R1067Q variant, predicted to reside in the hydrophobic core of the LRRK2 LRR domain, may alter the electronic surface, domain stability, and interactions with other proteins [44]. Additionally, the LRRK2^R1067Q^ protein was found to be retained within cytosolic pools and strongly enhanced LRRK2-mediated pRab10^Thr73^ substrate phosphorylation [42]. This variant exhibited approximately a two-fold increase in LRRK2 kinase activity compared to the wild type, surpassing that of the p.G2019S variant [31]. Most reported p.R1067Q probands (15/19, 78.95%) lacked family histories, reflecting the incomplete penetrance of pathogenic *LRRK2* variants [23–31]. The PD patient carrying p.R1067Q in our cohort exhibited early-onset PD with typical PD symptoms, which aligns with those in reported *LRRK2*-p.R1067Q PD patients [31]. Interestingly, this finding is corroborated by data from MDSGene, showing early-onset PD in approximately one-third of patients with pathogenic or likely pathogenic *LRRK2* variants, which further supports the pathogenicity of p.R1067Q [20]. The p.D1756Y variant, affecting the conserved residue localized in the COR domain, may induce alterations in GTPase activity directly or indirectly, since disease-associated variants p.N1437H, p.R1441C/G/H/S, p.A1442P, p.V1447M, p.Y1699C, p.F1700L, and p.L1795F within ROC-COR have been reported to significantly stimulate kinase activity [20,40,42,45]. The homozygous p.D1756Y carrier (onset age: 77 years) showed slightly slower disease progression, consistent with other homozygous *LRRK2* patients, such as p.G2019S carriers, who did not exhibit worse brain pathology or severe phenotypes compared to heterozygotes [46,47]. Given that the heterozygous p.D1756Y variant has been previously reported, and no monogenic dosage effect has been established for homozygous *LRRK2* pathogenic variants, such as p.R1441H and p.G2019S, we propose that homozygous *LRRK2* variants like p.D1756Y may increase disease risk, particularly for those variant(s) with incomplete penetrance, though parental genotypes for co-segregation analysis were not available in this case [46–49].

Two *LRRK2* variants, p.A419V and p.G2385R, which exhibit higher prevalence and are associated with increased kinase activity but lack sufficient evidence for disease segregation, are recognized as genetic risk factors for PD [42,50,51]. The p.A419V variant, located in the conserved ARM domain, has been reported as a risk variant for PD in Chinese populations, with the allelic frequency up to 0.018 in PD cases (S2 Table) [52,53]. It can be supposed that the frequencies and pathogenicity of p.A419V are highly population-specific, with potentially stronger effects in the East Asian population, highlighting the importance of tracing founder haplotypes [20]. In this study, p.A419V was estimated to increase PD risk in Han Chinese by approximately four-fold, which is similar to the study by Li et al [52]. Several meta-analyses have also shown that p.A419V was significantly associated with higher PD risk in East Asians, though conflicting results were obtained from several case-control studies [50,53,54]. The average onset age of the PD patients in the positive studies appeared younger (<55 years), while negative studies were characterized by enrolled patients with a later onset age (>60 years) [55–57]. The significant association observed between p.A419V and early-onset PD but absence of an association with late-onset PD, suggested that this variant may moderately increase the PD susceptibility, though its impact appeared to be relatively limited [58,59]. The reduced penetrance in late-onset PD patients may be explained by the coexistence of protective factors or other confounding variables. Consistent with other reported *LRRK2* variants that stimulated LRRK2 kinase activity and suppressed LRRK2 biomarker phosphorylation, the p.A419V variant increased LRRK2 kinase activity and was associated with reduced phosphorylation at the biomarker site p.S955 [42].

Regarding the p.G2385R variant, structurally, replacing the hydrophobic glycine with a positively charged hydrophilic arginine at position 2385 could likely increase the net positive charge, inducing alterations in electrostatic repulsion and surface complementarity, therefore, it may interfere with the strength and quality of intra- and/or inter-molecular interactions [39,60,61]. The p.G2385R variant, located in the WD40 domain, has been shown to compromise WD40 homodimerization and lead to altered kinase activity in vitro [37,62]. The LRRK2^G2385R^ protein was more toxic and led to higher cell death and apoptosis intensity than wild-type protein under oxidative stress conditions [60]. Additionally, the p.G2385R variant led to decreased binding affinity of LRRK2 to synaptic vesicles and increased affinity to proteins involved in proteasomal degradation, associated with lower steady-state intracellular LRRK2 levels [61,63]. LRRK2^G2385R^ can cause elevated LRRK2 kinase activity in the human brain, leading to Lewy body pathology [64]. Multiple case-control studies and meta-analyses found evidence supporting the significant association between the p.G2385R variant and increased PD risk [65–72]. In our independent cohort of Han Chinese individuals, the p.G2385R variant was observed at allelic frequencies of 0.0438 in cases and of 0.0212 in controls, with an OR of 2.1149, in line with frequencies in other reported Chinese populations (0.0029–0.0711 in PD *vs.* 0–0.0355 in controls, S3 Table) [67,68,73,74]. Combining the data, the p.G2385R variant rare in Caucasian populations appeared to be an ethnic-specific genetic PD risk factor originating from Chinese ancestors around 4,800 years ago [24,60].

Several limitations of our study should be acknowledged. i) The main limitation of this study is its relatively modest sample size. Although it provided sufficient power (>0.7) to detect associations for the p.A419V and p.G2385R variants, the study had limited statistical power to detect associations involving rare variants or variants with more moderate effects. ii) Replication of the identified *LRRK2* variants in additional cohorts and the relevant functional validation are warranted to confirm their pathogenesis. We also envisage the collaborations with large-scale East Asian databases for validation. Furthermore, exploring the finer haplotype *LRRK2* structure and gene-environment interactions will offer a more comprehensive understanding of its contribution to PD. Advances in variant detection and functional evidence will drive the re-classification of specific *LRRK2* variants in resources like MDSGene, thereby paving the way for improved genetic counseling and personalized therapeutic strategies for PD [20].

## Conclusion

In summary, the *LRRK2* variants p.R1067Q and p.D1756Y might play a likely pathogenic role without full penetrance in PD, while the variants p.A419V and p.G2385R might be risk factors for increased PD susceptibility in the Han Chinese population. These findings rooted in Han Chinese will help bridge the significant gap in knowledge regarding genetic variants across different populations and improve the global generalizability of PD genetic risk score predictions. Our systematic and comprehensive *LRRK2* variant analysis may provide valuable variant markers, optimize future genetic testing and patient-centric genetic counseling, inform the development of cause-targeted therapies, and promote the preclinical development of small molecule kinase inhibitors.

## Supporting information

S1 Fig Sanger sequencing chromatograms confirming heterozygous or homozygous *LRRK2* variants in PD patients (A-M), and the wild-type *LRRK2* sequence in controls (N-Y). (PDF)

S1 Table Primers used for the identification of *LRRK2* variants. (PDF)

S2 Table Distribution of the *LRRK2* p.A419V variant between PD cases and controls in different countries/regions, and its association with PD. (PDF)

S3 Table Distribution of the *LRRK2* p.G2385R variant between PD cases and controls in different countries/regions, and its association with PD. (PDF)
