# Supplementary material for: Genetic analysis of LRRK2 variants in Han Chinese patients with Parkinson’s disease
Source: PLoS One. 2026 Jan 8;21(1):e0340448. doi: 10.1371/journal.pone.0340448 (PMC12782381; doi:10.1371/journal.pone.0340448)

S1 Fig. Sanger sequencing chromatograms confirming heterozygous or homozygous *LRRK2* variants in PD patients (A-M), and the wild-type *LRRK2* sequence in controls (N-Y).

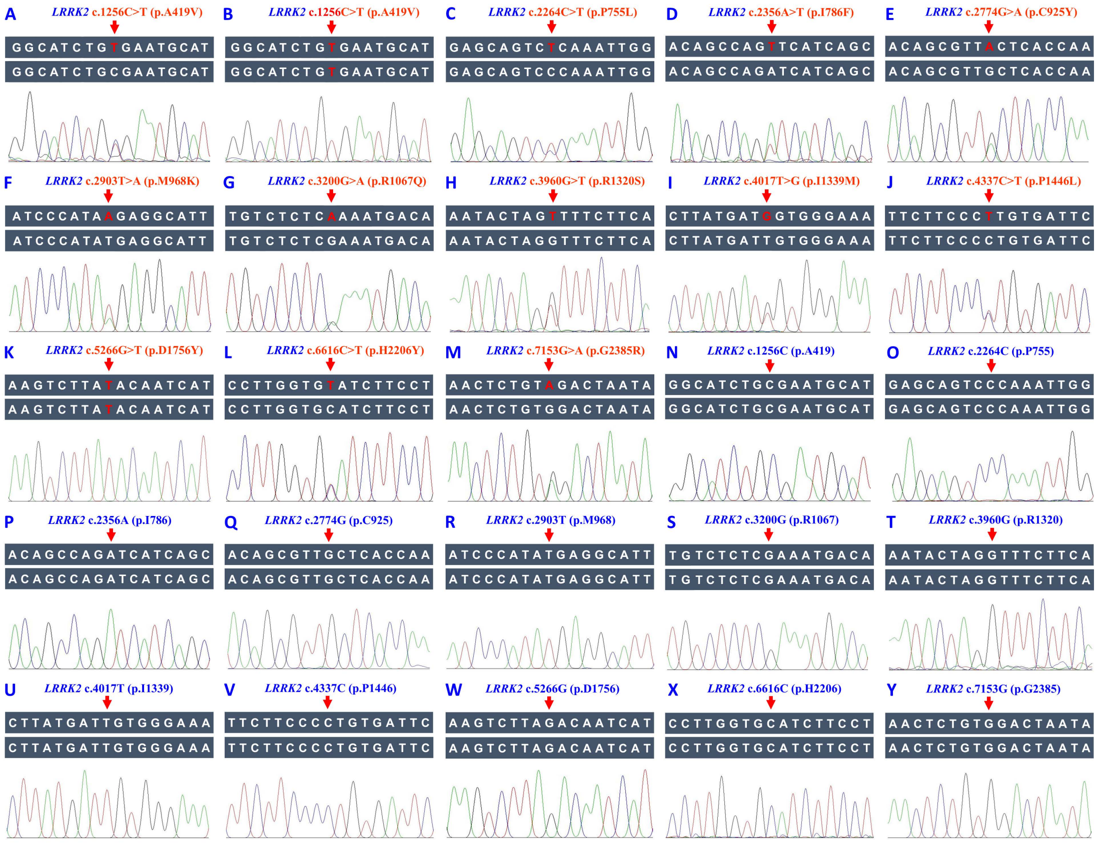

Supplement: S1 Fig — (PDF) [file pone.0340448.s001.pdf]
