# Supplementary material for: Genetic analysis of LRRK2 variants in Han Chinese patients with Parkinson’s disease
Source: PLoS One. 2026 Jan 8;21(1):e0340448. doi: 10.1371/journal.pone.0340448 (PMC12782381; doi:10.1371/journal.pone.0340448)
Supplement: S1 Table — (PDF) [file pone.0340448.s002.pdf]

S1 Table. Primers used for the identification of *LRRK2* variants.

Primer name	Primer sequence (5'→3')	Product size (bp)
LRRK2-c.1256C>T-F	TCCCAGCTCATAGGGAAGTG	153
LRRK2-c.1256C>T-R	CCACAAAAATGTGTTTCCCTTT	
LRRK2-c.2264C>T-F	TTGCCAGTCTCCTAAAAGGAA	176
LRRK2-c.2264C>T-R	CTGTCACCTTTCCCAATGCT	
LRRK2-c.2356A>T-F	TCCCAAATTGGTGGAACTCT	200
LRRK2-c.2356A>T-R	AAGGACCAAGCCAAGAAGGT	
LRRK2-c.2774G>A-F	GGAAGTGAAGGCTCATTTCTTG	229
LRRK2-c.2774G>A-R	CATCAGGGAAATCCCTACCA	
LRRK2-c.2903T>A-F	TGCCTTGAAGAAAGCCTGAT	238
LRRK2-c.2903T>A-R	CATTTCTGGCTTAGGGCATC	
LRRK2-c.3200G>A-F	TTGACACATTTGGACTTGCAC	226
LRRK2-c.3200G>A-R	GCTGCTCCAGTTTCTCTACCA	
LRRK2-c.3960G>T/c.4017T>G-F	TGTGACATGTAAAAGAACTCACCT	231
LRRK2-c.3960G>T/c.4017T>G-R	CAACTGTGGCACTTTGCATT	
LRRK2-c.4337C>T-F	TGAATGTCACGGAAAGCAAA	209
LRRK2-c.4337C>T-R	GAACCCTCGCTTATTCAGGA	
LRRK2-c.5266G>T-F	CACTTGTGTTGTGTGCAGTAGA	345
LRRK2-c.5266G>T-R	ACAGAGCTCAACACCTTGTCT	
LRRK2-c.6616C>T-F	TGCAAGAAAGCAAAAAGAGTT	225
LRRK2-c.6616C>T-R	TTCGGTATTGATGACCAGGAG	
LRRK2-c.7153G>A-F	TGCAGCTTTCAGTGATTCCA	233
LRRK2-c.7153G>A-R	GGCAGAAAGGAAGAAAAATCC	

bp, base pairs; F, forward sequence; *LRRK2*, the leucine rich repeat kinase 2 gene; R, reverse sequence.
