# Supplementary material for: Genetic analysis of LRRK2 variants in Han Chinese patients with Parkinson’s disease
Source: PLoS One. 2026 Jan 8;21(1):e0340448. doi: 10.1371/journal.pone.0340448 (PMC12782381; doi:10.1371/journal.pone.0340448)
Supplement: S2 Table — (PDF) [file pone.0340448.s003.pdf]

S2 Table. Distribution of the *LRRK2* p.A419V variant between PD cases and controls in different countries/regions, and its association with PD.

Country/ region	References	PD cases and controls	Variant carriers (proportion)				Minor alleles (frequency)			
			PD	Controls	*P* value	OR (95% CI)	PD	Controls	*P* value	OR (95% CI)
Arab-Berber	Ross et al., 2011 [1]	240/372	0	0	-	-	0	0	-	-
Asia	Gopalai et al., 2013 [2]	404/424[a]	1 (0.25%)	3 (0.71%)	NR	NR	1 (0.0012)	3 (0.0035)	0.625	0.35 (0.04–3.38)
	Foo et al., 2014 [3]	375/399[b]	14 (3.73%)	6 (1.50%)	NR	NR	NR	NR	NR	NR
Caucasian	Ross et al., 2011 [1]	6,995/5,595	5 (0.07%)	3 (0.05%)	NR	NR	NR	NR	NR	NR
Central Europe	Skorvanek et al., 2021 [4]	726/340	2 (0.28%)	0	NR	NR	2 (0.0014)	0	NR	NR
China	Di Fonzo et al., 2006 [5]	592/344[c]	10 (1.69%)	3 (0.87%)	>0.05	NR	10 (0.0084)	3 (0.0044)	>0.05	NR
	Li et al., 2012 [6]	729/585[d]	22 (3.02%)	4 (0.68%)	0.003	4.14 (1.53–12.74)	22 (0.0151)	4 (0.0034)	NR	NR
	Wu et al., 2012 [7]	472/443	NR	NR	NR	NR	5 (0.0053)	3 (0.0034)	0.56	1.56 (0.37–6.5)
	Wu et al., 2012 [7]	485/494[c]	NR	NR	NR	NR	4 (0.0041)	6 (0.0061)	0.57	0.68 (0.19–2.41)
	Ross et al., 2011; Heckman et al., 2013 [1,8]	369/300[c]	NR	NR	NR	NR	NR (0.0137)	NR (0.0017)	0.057	7.51 (0.95–59.60)
	Wu-Chou et al., 2013 [9]	626/473[c,d]	0	0	-	-	0	0	-	-

Country/ region	References	PD cases and controls	Variant carriers (proportion)				Minor alleles (frequency)			
			PD	Controls	*P* value	OR (95% CI)	PD	Controls	*P* value	OR (95% CI)
	Li et al., 2015 [10]	500/574	18 (3.60%)	9 (1.57%)	0.025	2.57 (1.13–5.86)	18 (0.0180)	9 (0.0078)	0.002	2.57 (1.43–4.60)
	Zhang et al., 2018 [11]	296/643[d]	6 (2.03%)	7 (1.09%)	NR	NR	NR	NR	NR	NR
	Zheng et al., 2020 [12]	191/200	8 (4.19%)	0	NR	NR	NR	0	NR	NR
Japan	Ross et al., 2011; Heckman et al., 2013 [1,8]	177/98	NR	NR	NR	NR	NR (0.0263)	NR (0.0211)	0.70	1.26 (0.38–4.22)
Kazakhstan	Kaiyrzhanov et al., 2020 [13]	242/199	9 (3.72%)	5 (2.51%)	0.4	1.5 (0.49–4.55)	10 (0.0207)	5 (0.0126)	NR	NR
Nigeria	Rizig et al., 2021 [14]	92/210	0	0	-	-	0	0	-	-
Russia	Usenko et al., 2023 [15]	508/470	7 (1.38%)	3 (0.64%)	0.38	1.84 (0.47–7.14)	NR	NR	NR	NR
Singapore	Tan et al., 2010 [16]	250/250[d]	0	0	-	-	0	0	-	-
	Wu et al., 2012 [7]	560/550	NR	NR	NR	NR	4 (0.0036)	4 (0.0036)	0.98	0.98 (0.24–3.93)
South Korea	Ross et al., 2011; Heckman et al., 2013 [1,8]	830/564	NR	NR	NR	NR	NR (0.0294)	NR (0.0126)	0.011	2.21 (1.20–4.06)
Tunisia	Jasinska-Myga et al., 2010 [17]	165/364[e]	0	0	-	-	0	0	-	-

LRRK2, the leucine rich repeat kinase 2 gene; NR, not reported; OR (95% CI), odds ratio with 95% confidence interval; PD, Parkinson's disease.

[a]The subjects were of Chinese, Malay, or Indian ethnicity (223 Chinese, 122 Malays, and 59 Indians in 404 PD cases; 236 Chinese, 110 Malays, and 78 Indians in 424 controls), and all p.A419V variant carriers were Chinese.

[b]The subjects were of Chinese or Korean ethnicity (195 Chinese and 180 Koreans in 375 PD cases; 219 Chinese and 180 Koreans in 399 controls).

[c]The subjects were from Taiwan, China.

[d]The subjects were of Han Chinese.

[e]The subjects were of Arab-Berber ethnicity.

References

1. Ross OA, Soto-Ortolaza AI, Heckman MG, Aasly JO, Abahuni N, Annesi G, et al. Association of LRRK2 exonic variants with susceptibility to Parkinson's disease: a case-control study. Lancet Neurol. 2011;10(10):898–908. https://doi.org/10.1016/S1474-4422(11)70175-2 PMID: 21885347
2. Gopalai AA, Lim SY, Aziz ZA, Lim SK, Tan LP, Chong YB, et al. Lack of association between the LRRK2 A419V variant and Asian Parkinson's disease. Ann Acad Med Singap. 2013;42(5):237–40. https://doi.org/10.47102/annals-acadmedsg.V42N5p237 PMID: 23771111
3. Foo JN, Tan LC, Liany H, Koh TH, Irwan ID, Ng YY, et al. Analysis of non-synonymous-coding variants of Parkinson's disease-related pathogenic and susceptibility genes in East Asian populations. Hum Mol Genet. 2014;23(14):3891–7. https://doi.org/10.1093/hmg/ddu086 PMID: 24565865
4. Skorvanek M, Rizig M, Athanasiou-Fragkouli A, Necpal J, Straka I, Tamas G, et al. LRRK2 mutations in Parkinson's disease patients from Central Europe: a case control study. Parkinsonism Relat Disord. 2021;83:110–2. https://doi.org/10.1016/j.parkreldis.2020.12.021 PMID: 33561776
5. Di Fonzo A, Wu-Chou YH, Lu CS, van Doeselaar M, Simons EJ, Rohé CF, et al. A common missense variant in the LRRK2 gene, Gly2385Arg, associated with Parkinson's disease risk in Taiwan. Neurogenetics. 2006;7(3):133–8. https://doi.org/10.1007/s10048-006-0041-5 PMID: 16633828
6. Li NN, Tan EK, Chang XL, Mao XY, Zhang JH, Zhao DM, et al. Genetic analysis of LRRK2 A419V variant in ethnic Chinese. Neurobiol Aging. 2012;33(8):1849.e1–3. https://doi.org/10.1016/j.neurobiolaging.2012.02.013 PMID: 22418733
7. Wu YR, Tan LC, Fu X, Chen CM, Au WL, Chen L, et al. LRRK2 A419V is not associated with Parkinson's disease in different Chinese populations. PLoS One. 2012;7(7):e36123. https://doi.org/10.1371/journal.pone.0036123 PMID: 22807999

8. Heckman MG, Soto-Ortolaza AI, Aasly JO, Abahuni N, Annesi G, Bacon JA, et al. Population-specific frequencies for LRRK2 susceptibility variants in the Genetic Epidemiology of Parkinson's Disease (GEO-PD) Consortium. Mov Disord. 2013;28(12):1740–4. https://doi.org/10.1002/mds.25600 PMID: 23913756
9. Wu-Chou YH, Chen YT, Yeh TH, Chang HC, Weng YH, Lai SC, et al. Genetic variants of SNCA and LRRK2 genes are associated with sporadic PD susceptibility: a replication study in a Taiwanese cohort. Parkinsonism Relat Disord. 2013;19(2):251–5. https://doi.org/10.1016/j.parkreldis.2012.10.019 PMID: 23182315
10. Li K, Tang BS, Liu ZH, Kang JF, Zhang Y, Shen L, et al. LRRK2 A419V variant is a risk factor for Parkinson's disease in Asian population. Neurobiol Aging. 2015;36(10):2908.e11–5. https://doi.org/10.1016/j.neurobiolaging.2015.07.012 PMID: 26234753
11. Zhang JR, Jin H, Li K, Mao CJ, Yang YP, Wang F, et al. Genetic analysis of LRRK2 in Parkinson's disease in Han Chinese population. Neurobiol Aging. 2018;72:187.e5–10. https://doi.org/10.1016/j.neurobiolaging.2018.06.036 PMID: 30049590
12. Zheng R, Jin CY, Chen Y, Ruan Y, Gao T, Lin ZH, et al. Analysis of rare variants of autosomal-dominant genes in a Chinese population with sporadic Parkinson's disease. Mol Genet Genomic Med. 2020;8(10):e1449. https://doi.org/10.1002/mgg3.1449 PMID: 32794657
13. Kaiyrzhanov R, Aitkulova A, Shashkin C, Zharkinbekova N, Rizig M, Zholdybayeva E, et al. LRRK2 mutations and Asian disease-associated variants in the first Parkinson's disease cohort from Kazakhstan. Parkinsons Dis. 2020;2020:2763838. https://doi.org/10.1155/2020/2763838 PMID: 32148752
14. Rizig M, Ojo OO, Athanasiou-Fragkouli A, Agabi OP, Oshinaike OO, Houlden H, et al. Negative screening for 12 rare LRRK2 pathogenic variants in a cohort of Nigerians with Parkinson's disease. Neurobiol Aging. 2021;99:101.e15–9. https://doi.org/10.1016/j.neurobiolaging.2020.09.024 PMID: 33158606
15. Usenko TS, Senkevich KA, Basharova KS, Bezrukova AI, Baydakova GV, Tyurin AA, et al. LRRK2 exonic variants are associated with lysosomal hydrolase activities and lysosphingolipid alterations in Parkinson's disease. Gene. 2023;882:147639. https://doi.org/10.1016/j.gene.2023.147639 PMID: 37473971.
16. Tan EK, Peng R, Teo YY, Tan LC, Angeles D, Ho P, et al. Multiple LRRK2 variants modulate risk of Parkinson disease: a Chinese multicenter study. Hum Mutat. 2010;31(5):561–8. https://doi.org/10.1002/humu.21225 PMID: 20186690
17. Jasinska-Myga B, Kachergus J, Vilariño-Güell C, Wider C, Soto-Ortolaza AI, Kefi M, et al. Comprehensive sequencing of the LRRK2 gene in patients with familial Parkinson's disease from North Africa. Mov Disord. 2010;25(13):2052–8. https://doi.org/10.1002/mds.23283 PMID: 20721913
