# Supplementary material for: Genetic analysis of LRRK2 variants in Han Chinese patients with Parkinson’s disease
Source: PLoS One. 2026 Jan 8;21(1):e0340448. doi: 10.1371/journal.pone.0340448 (PMC12782381; doi:10.1371/journal.pone.0340448)
Supplement: S3 Table — (PDF) [file pone.0340448.s004.pdf]

S3 Table. Distribution of the *LRRK2* p.G2385R variant between PD cases and controls in different countries/regions, and its association with PD.

Country/ region	Author (year)	PD cases and controls	Variant carriers (proportion)				Minor alleles (frequency)			
			PD	Controls	*P* value	OR (95% CI)	PD	Controls	*P* value	OR (95% CI)
Arab-Berber	Ross et al., 2011 [1]	240/372	0	0	-	-	0	0	-	-
Asia	Ross et al., 2011 [1]	1,376/962	NR	NR	NR	NR	NR	NR	0.0026	1.73 (1.20–2.49)
	Foo et al., 2014 [2]	375/399[a]	29 (7.73%)	16 (4.01%)	NR	NR	NR	NR	NR	NR
Austria	Haubenberger et al., 2007 [3]	162/288	0	0	-	-	0	0	-	-
Caucasian	Ross et al., 2011 [1]	6,995/5,595	0	0	-	-	0	0	-	-
Central Europe	Skorvanek et al., 2021 [4]	731/342	0	0	-	-	0	0	-	-
China	Di Fonzo et al., 2006 [5]	608/373[b]	61 (10.03%)	18 (4.83%)	0.012	2.24 (1.29–3.88)	61 (0.0502)	18 (0.0241)	0.012	NR
	Fung et al., 2006 [6]	305/176[b,c]	27 (8.85%)	1 (0.57%)	0.0002	16.99 (2.29–126.21)	27 (0.0443)	1 (0.0028)	NR	NR
	Li et al., 2007 [7]	235/214[c]	14 (5.96%)	0	<0.05	NR	14 (0.0298)	0	NR	NR
	An et al., 2008 [8]	600/334[c]	71 (11.83%)	11 (3.29%)	<0.01	3.94 (2.06–7.55)	72 (0.0600)	11 (0.0165)	NR	NR
	Tan et al., 2010 [9]	628/510[c]	NR	NR	NR	NR	NR (0.06)	NR (0.02)	0.000055	3.64 (1.86–7.10)
	Tan et al., 2010 [9]	293/299[b,c]	NR	NR	NR	NR	NR (0.05)	NR (0.02)	0.006	2.53 (1.28–5.00)
	Lin et al., 2011 [10]	452/288[b]	33 (7.30%)	13 (4.51%)	0.03	NR	34 (0.0376)	13 (0.0226)	NR	NR
	Liu et al., 2012 [11]	464/549	64 (13.79%)	26 (4.74%)	<0.0001	NR	66 (0.0711)	26 (0.0237)	<0.0001	NR
	Wang et al., 2012 [12]	2,013/1,971[c]	NR	NR	<0.0001	1.63 (1.30–2.05)	231 (0.0574)	140 (0.0355)	<0.0001	1.65 (1.33–2.05)

Country/ region	Author (year)	PD cases and controls	Variant carriers (proportion)				Minor alleles (frequency)			
			PD	Controls	*P* value	OR (95% CI)	PD	Controls	*P* value	OR (95% CI)
	Yan et al., 2012 [13]	183/180[c]	20 (10.93%)	6 (3.33%)	0.005	NR	20 (0.0546)	6 (0.0167)	0.006	3.41 (1.42–8.19)
	Yan et al., 2012 [13]	171/160[d]	1 (0.58%)	1 (0.63%)	0.962	NR	1 (0.0029)	1 (0.0031)	0.962	NR
	Zhou et al., 2012 [14]	202/212[c]	26 (12.87%)	12 (5.66%)	0.015	2.26 (1.15–4.45)	27 (0.0668)	13 (0.0307)	NR	NR
	Cai et al., 2013 [15]	510/550[c]	49 (9.61%)	12 (2.18%)	NR	NR	49 (0.0480)	12 (0.0109)	<0.01	4.58 (2.42–8.65)
	Fu et al., 2013 [16]	446/403[c]	37 (8.30%)	16 (3.97%)	0.012	2.13 (1.17–3.91)	38 (0.0426)	16 (0.0199)	NR	NR
	Ma et al., 2013 [17]	237/190[c]	24 (10.13%)	4 (2.11%)	0.009	5.24 (1.79–15.38)	24 (0.0506)	4 (0.0105)	0.090	5.01 (1.72–14.58)
	Wu et al., 2013 [18]	573/503[b]	NR (9.2%)	NR (4.2%)	0.0014	2.34 (1.41–4.02)	NR (0.046)	NR (0.021)	0.0017	2.27 (1.38–3.88)
	Wu-Chou et al., 2013 [19]	941/618[b,c]	78 (8.29%)	37 (5.99%)	NR	NR	78 (0.0414)	37 (0.0299)	0.089	1.40 (0.9411–2.0861)
	Dan et al., 2014 [20]	561/556[c]	53 (9.45%)	29 (5.22%)	0.014	1.86 (1.08–3.19)	56 (0.0499)	29 (0.0261)	0.004	NR
	Zhang et al., 2018 [21]	296/643[c]	55 (18.58%)	50 (7.78%)	<0.001	2.71 (1.79–4.08)	NR	NR	NR	NR
	Liu et al., 2020 [22]	158/275[c]	NR	0	NR	NR	NR (0.0475)	0	<0.001	NR
	Wu et al., 2020 [23]	260/271[c]	29 (11.15%)	15 (5.54%)	0.021	2.14 (1.12–4.10)	30 (0.0577)	16 (0.0295)	0.023	2.05 (1.11–3.82)
Iran	Shojaee et al., 2009 [24]	205/200	0	0	-	-	0	0	-	-
Japan	Funayama et al., 2007 [25]	448/457	52 (11.61%)	22 (4.81%)	NR	NR	54 (0.0603)	22 (0.0241)	0.000124	2.63 (1.56–4.35)
	Zabetian et al., 2009 [26]	601/1,628	69 (11.48%)	101 (6.20%)	0.00033	1.83 (1.31–2.54)	NR	NR	NR	NR
	Seki et al., 2011 [27]	100[e]/233	18 (18.00%)	10 (4.29%)	NR	NR	18 (0.0900)	10 (0.0215)	0.0000534	NR

Country/ region	Author (year)	PD cases and controls	Variant carriers (proportion)				Minor alleles (frequency)			
			PD	Controls	*P* value	OR (95% CI)	PD	Controls	*P* value	OR (95% CI)
	Seki et al., 2011 [27]	73[f]/233	5 (6.85%)	10 (4.29%)	NR	NR	5 (0.0342)	10 (0.0215)	0.38	NR
	Li et al., 2020 [28]	1,402/216	146 (10.41%)	11 (5.09%)	NR	NR	150 (0.0535)	11 (0.0255)	0.013	2.16 (1.16–4.02)
Kazakhstan	Kaiyrzhanov et al., 2020 [29]	239/199	3 (1.26%)	2 (1.01%)	NR	NR	3 (0.0063)	2 (0.0050)	0.8	1.25 (0.21–7.57)
Malaysia	Gopalai et al., 2014 [30]	695/507	NR	NR	NR	NR	36 (0.0259)	12 (0.0118)	0.019	2.22 (1.15–4.29)
Nigeria	Rizig et al., 2021 [31]	92/210	0	0	-	-	0	0	-	-
Norway	Toft et al., 2007 [32]	433/587	0	0	-	-	0	0	-	-
Russia	Usenko et al., 2023 [33]	508/470	1 (0.20%)	0	0.60	2.34 (0.10–57.64)	NR	NR	NR	NR
Singapore	Tan et al., 2010 [9]	250/250[c]	NR	NR	NR	NR	NR (0.04)	NR (0.02)	0.03	2.34 (1.06–5.17)
	Tan et al., 2010 [9]	192/192[c]	NR	NR	NR	NR	NR (0.06)	NR (0.01)	0.002	4.34 (1.62–11.65)
	Tan et al., 2007 [34]	166/306[g]	2 (1.20%)	2 (0.65%)	0.3	2.83 (0.40–20.2)	2 (0.0060)	2 (0.0033)	NR	NR
	Tan et al., 2007 [35]	495/494[h]	37 (7.47%)	18 (3.64%)	0.002	2.67 (1.43–4.99)	38 (0.0384)	18 (0.0182)	NR	NR
South Korea	Choi et al., 2008 [36]	72/100	9 (12.50%)	5 (5.00%)	0.09	2.71 (0.87–8.48)	NR	NR	NR	NR
	Kim et al., 2010 [37]	923/422	82 (8.88%)	21 (4.98%)	0.0170	1.83 (1.11–3.00)	84 (0.0455)	21 (0.0249)	0.0104	NR

LRRK2, the leucine rich repeat kinase 2 gene; NR, not reported; OR (95% CI), odds ratio with 95% confidence interval; PD, Parkinson's disease.
[a]The subjects were of Chinese or Korean ethnicity (195 Chinese and 180 Koreans in 375 PD cases; 219 Chinese and 180 Koreans in 399 controls).
[b]The subjects were from Taiwan, China.
[c]The subjects were of Han Chinese.
[d]The subjects were of Uyghur origin.
[e]The patients were from autosomal dominant PD families.
[f]The patients were sporadic PD.

[g]The subjects were non-Chinese Asians of Malay or Indian ethnicity.
[h]The subjects were of Chinese ethnicity.

References

1. Ross OA, Soto-Ortolaza AI, Heckman MG, Aasly JO, Abahuni N, Annesi G, et al. Association of LRRK2 exonic variants with susceptibility to Parkinson's disease: a case-control study. Lancet Neurol. 2011;10(10):898–908. https://doi.org/10.1016/S1474-4422(11)70175-2 PMID: 21885347
2. Foo JN, Tan LC, Liany H, Koh TH, Irwan ID, Ng YY, et al. Analysis of non-synonymous-coding variants of Parkinson's disease-related pathogenic and susceptibility genes in East Asian populations. Hum Mol Genet. 2014;23(14):3891–7. https://doi.org/10.1093/hmg/ddu086 PMID: 24565865
3. Haubenberger D, Bonelli S, Hotzy C, Leitner P, Lichtner P, Samal D, et al. A novel LRRK2 mutation in an Austrian cohort of patients with Parkinson's disease. Mov Disord. 2007;22(11):1640–3. https://doi.org/10.1002/mds.21568 PMID: 17523199
4. Skorvanek M, Rizig M, Athanasiou-Fragkouli A, Necpal J, Straka I, Tamas G, et al. LRRK2 mutations in Parkinson's disease patients from Central Europe: a case control study. Parkinsonism Relat Disord. 2021;83:110–2. https://doi.org/10.1016/j.parkreldis.2020.12.021 PMID: 33561776
5. Di Fonzo A, Wu-Chou YH, Lu CS, van Doeselaar M, Simons EJ, Rohé CF, et al. A common missense variant in the LRRK2 gene, Gly2385Arg, associated with Parkinson's disease risk in Taiwan. Neurogenetics. 2006;7(3):133–8. https://doi.org/10.1007/s10048-006-0041-5 PMID: 16633828
6. Fung HC, Chen CM, Hardy J, Singleton AB, Wu YR. A common genetic factor for Parkinson disease in ethnic Chinese population in Taiwan. BMC Neurol. 2006;6:47. https://doi.org/10.1186/1471-2377-6-47 PMID: 17187665
7. Li C, Ting Z, Qin X, Ying W, Li B, Guo Qiang L, et al. The prevalence of LRRK2 Gly2385Arg variant in Chinese Han population with Parkinson's disease. Mov Disord. 2007;22(16):2439–43. https://doi.org/10.1002/mds.21763 PMID: 17960808
8. An XK, Peng R, Li T, Burgunder JM, Wu Y, Chen WJ, et al. LRRK2 Gly2385Arg variant is a risk factor of Parkinson's disease among Han-Chinese from mainland China. Eur J Neurol. 2008;15(3):301–5. https://doi.org/10.1111/j.1468-1331.2007.02052.x PMID: 18201193
9. Tan EK, Peng R, Teo YY, Tan LC, Angeles D, Ho P, et al. Multiple LRRK2 variants modulate risk of Parkinson disease: a Chinese multicenter study. Hum Mutat. 2010;31(5):561–8. https://doi.org/10.1002/humu.21225 PMID: 20186690
10. Lin CH, Wu RM, Tai CH, Chen ML, Hu FC. Lrrk2 S1647T and BDNF V66M interact with environmental factors to increase risk of Parkinson's disease. Parkinsonism Relat Disord. 2011;17(2):84–8. https://doi.org/10.1016/j.parkreldis.2010.11.011 PMID: 21167764
11. Liu J, Zhou Y, Wang C, Wang T, Zheng Z, Chan P. Brain-derived neurotrophic factor (BDNF) genetic polymorphism greatly

increases risk of leucine-rich repeat kinase 2 (LRRK2) for Parkinson's disease. Parkinsonism Relat Disord. 2012;18(2):140–3. https://doi.org/10.1016/j.parkreldis.2011.09.002 PMID: 21924942

12. Wang C, Cai Y, Zheng Z, Tang BS, Xu Y, Wang T, et al. Penetrance of LRRK2 G2385R and R1628P is modified by common PD-associated genetic variants. Parkinsonism Relat Disord. 2012;18(8):958–63. https://doi.org/10.1016/j.parkreldis.2012.05.003 PMID: 22658533
13. Yan H, Ma Q, Yang X, Wang Y, Yao Y, Li H. Correlation between LRRK2 gene G2385R polymorphisms and Parkinson's disease. Mol Med Rep. 2012;6(4):879–83. https://doi.org/10.3892/mmr.2012.1008 PMID: 22842796
14. Zhou Y, Luo X, Li F, Tian X, Zhu L, Yang Y, et al. Association of Parkinson's disease with six single nucleotide polymorphisms located in four PARK genes in the northern Han Chinese population. J Clin Neurosci. 2012;19(7):1011–5. https://doi.org/10.1016/j.jocn.2011.09.028 PMID: 22575062
15. Cai J, Lin Y, Chen W, Lin Q, Cai B, Wang N, et al. Association between G2385R and R1628P polymorphism of LRRK2 gene and sporadic Parkinson's disease in a Han-Chinese population in south-eastern China. Neurol Sci. 2013;34(11):2001–6. https://doi.org/10.1007/s10072-013-1436-3 PMID: 23624603
16. Fu X, Zheng Y, Hong H, He Y, Zhou S, Guo C, et al. LRRK2 G2385R and LRRK2 R1628P increase risk of Parkinson's disease in a Han Chinese population from Southern Mainland China. Parkinsonism Relat Disord. 2013;19(3):397–8. https://doi.org/10.1016/j.parkreldis.2012.08.007 PMID: 22981185
17. Ma QL, An XK, Li ZM, Zhang HJ, Huang WQ, Cai LL, et al. P268S in NOD2 associates with susceptibility to Parkinson's disease in Chinese population. Behav Brain Funct. 2013;9:19. https://doi.org/10.1186/1744-9081-9-19. PMID: 23651603
18. Wu YR, Chang KH, Chang WT, Hsiao YC, Hsu HC, Jiang PR, et al. Genetic variants of LRRK2 in Taiwanese Parkinson's disease. PLoS One. 2013;8(12):e82001. https://doi.org/10.1371/journal.pone.0082001 PMID: 24339985
19. Wu-Chou YH, Chen YT, Yeh TH, Chang HC, Weng YH, Lai SC, et al. Genetic variants of SNCA and LRRK2 genes are associated with sporadic PD susceptibility: a replication study in a Taiwanese cohort. Parkinsonism Relat Disord. 2013;19(2):251–5. https://doi.org/10.1016/j.parkreldis.2012.10.019 PMID: 23182315
20. Dan X, Wang C, Ma J, Feng X, Wang T, Zheng Z, et al. MAPT IVS1+124 C>G modifies risk of LRRK2 G2385R for Parkinson's disease in Chinese individuals. Neurobiol Aging. 2014;35(7):1780.e7–10. https://doi.org/10.1016/j.neurobiolaging.2014.01.025 PMID: 24559644
21. Zhang JR, Jin H, Li K, Mao CJ, Yang YP, Wang F, et al. Genetic analysis of LRRK2 in Parkinson's disease in Han Chinese population. Neurobiol Aging. 2018;72:187.e5–10. https://doi.org/10.1016/j.neurobiolaging.2018.06.036 PMID: 30049590
22. Liu HJ, Li XY, Chen H, Yu HL, Tao QQ, Wu ZY. Identification of susceptibility loci for cognitive impairment in a cohort of Han Chinese patients with Parkinson's disease. Neurosci Lett. 2020;730:135034. https://doi.org/10.1016/j.neulet.2020.135034 PMID: 32404250

23. Wu Y, Pei Y, Yang Z, Li K, Lou X, Cui W. Accelerated telomere shortening independent of LRRK2 variants in Chinese patients with Parkinson's disease. Aging (Albany NY). 2020;12(20):20483–92. https://doi.org/10.18632/aging.103878 PMID: 33122450
24. Shojaee S, Sina F, Farboodi N, Fazlali Z, Ghazavi F, Ghorashi SA, et al. A clinic-based screening of mutations in exons 31, 34, 35, 41, and 48 of LRRK2 in Iranian Parkinson's disease patients. Mov Disord. 2009;24(7):1023–7. https://doi.org/10.1002/mds.22503 PMID: 19353692
25. Funayama M, Li Y, Tomiyama H, Yoshino H, Imamichi Y, Yamamoto M, et al. Leucine-rich repeat kinase 2 G2385R variant is a risk factor for Parkinson disease in Asian population. Neuroreport. 2007;18(3):273–5. https://doi.org/10.1097/WNR.0b013e32801254b6 PMID: 17314670
26. Zabetian CP, Yamamoto M, Lopez AN, Ujike H, Mata IF, Izumi Y, et al. LRRK2 mutations and risk variants in Japanese patients with Parkinson's disease. Mov Disord. 2009;24(7):1034–41. https://doi.org/10.1002/mds.22514 PMID: 19343804
27. Seki N, Takahashi Y, Tomiyama H, Rogaeva E, Murayama S, Mizuno Y, et al. Comprehensive mutational analysis of LRRK2 reveals variants supporting association with autosomal dominant Parkinson's disease. J Hum Genet. 2011;56(9):671–5. https://doi.org/10.1038/jhg.2011.79 PMID: 21796139
28. Li Y, Ikeda A, Yoshino H, Oyama G, Kitani M, Daida K, et al. Clinical characterization of patients with leucine-rich repeat kinase 2 genetic variants in Japan. J Hum Genet. 2020;65(9):771–81. https://doi.org/10.1038/s10038-020-0772-4 PMID: 32398759
29. Kaiyrzhanov R, Aitkulova A, Shashkin C, Zharkinbekova N, Rizig M, Zholdybayeva E, et al. LRRK2 mutations and Asian disease-associated variants in the first Parkinson's disease cohort from Kazakhstan. Parkinsons Dis. 2020;2020:2763838. https://doi.org/10.1155/2020/2763838 PMID: 32148752
30. Gopalai AA, Lim SY, Chua JY, Tey S, Lim TT, Mohamed Ibrahim N, et al. LRRK2 G2385R and R1628P mutations are associated with an increased risk of Parkinson's disease in the Malaysian population. Biomed Res Int. 2014;2014:867321. https://doi.org/10.1155/2014/867321 PMID: 25243190
31. Rizig M, Ojo OO, Athanasiou-Fragkouli A, Agabi OP, Oshinaike OO, Houlden H, et al. Negative screening for 12 rare LRRK2 pathogenic variants in a cohort of Nigerians with Parkinson's disease. Neurobiol Aging. 2021;99:101.e15–9. https://doi.org/10.1016/j.neurobiolaging.2020.09.024 PMID: 33158606
32. Toft M, Haugarvoll K, Ross OA, Farrer MJ, Aasly JO. LRRK2 and Parkinson's disease in Norway. Acta Neurol Scand Suppl. 2007;187:72–5. https://doi.org/10.1111/j.1600-0404.2007.00852.x. PMID: 17419834
33. Usenko TS, Senkevich KA, Basharova KS, Bezrukova AI, Baydakova GV, Tyurin AA, et al. LRRK2 exonic variants are associated with lysosomal hydrolase activities and lysosphingolipid alterations in Parkinson's disease. Gene. 2023;882:147639. https://doi.org/10.1016/j.gene.2023.147639 PMID: 37473971
34. Tan EK, Zhao Y, Tan L, Lim HQ, Lee J, Yuen Y, et al. Analysis of LRRK2 Gly2385Arg genetic variant in non-Chinese Asians. Mov Disord. 2007;22(12):1816–8. https://doi.org/10.1002/mds.21658 PMID: 17659642

35. Tan EK, Zhao Y, Skipper L, Tan MG, Di Fonzo A, Sun L, et al. The LRRK2 Gly2385Arg variant is associated with Parkinson's disease: genetic and functional evidence. Hum Genet. 2007;120(6):857–63. https://doi.org/10.1007/s00439-006-0268-0 PMID: 17019612
36. Choi JM, Woo MS, Ma HI, Kang SY, Sung YH, Yong SW, et al. Analysis of PARK genes in a Korean cohort of early-onset Parkinson disease. Neurogenetics. 2008;9(4):263–9. https://doi.org/10.1007/s10048-008-0138-0 PMID: 18704525
37. Kim JM, Lee JY, Kim HJ, Kim JS, Shin ES, Cho JH, et al. The LRRK2 G2385R variant is a risk factor for sporadic Parkinson's disease in the Korean population. Parkinsonism Relat Disord. 2010;16(2):85–8. https://doi.org/10.1016/j.parkreldis.2009.10.004 PMID: 19854095
